# Oral proteasome inhibitor with strong preclinical efficacy in myeloma models

**DOI:** 10.1186/s12885-016-2285-2

**Published:** 2016-03-24

**Authors:** Jonghoon Park, Eok Park, Cheol-Kyu Jung, Seung-Wan Kang, Byung Gyu Kim, Youngjoo Jung, Tae Hun Kim, Ji-Young Lim, Sung-Eun Lee, Chang-Ki Min, Kwang-Ai Won

**Affiliations:** R&D Center, LG Life Sciences, Ltd, Daejeon, South Korea; Department of Internal Medicine, Seoul St. Mary’s Hospital, The Catholic University of Korea, Seoul, South Korea

**Keywords:** Multiple myeloma, Proteasome inhibitor, Oral drug, Combination therapy, Pomalidomide

## Abstract

**Background:**

The proteasome is a validated anti-cancer target and various small-molecule inhibitors are currently in clinical development or on the market. However, adverse events and resistance associated with those proteasome inhibitors indicate the need for a new generation of drugs. Therefore, we focused on developing an oral proteasome inhibitor with improved efficacy and safety profiles.

**Method:**

The in vitro inhibition of the 20S proteasome catalytic activities was determined in human multiple myeloma (MM) cellular lysates with fluorogenic peptide substrates specific for each catalytic subunit. Cell cytotoxicity was assessed with the ATP bioluminescence assay using human cell samples from tumor cell lines, MM patients or normal healthy donors. In mice bearing human MM xenografts, a single dose of LC53-0110 was administered orally, and concentration-time profiles of LC53-0110 and the 20S proteasome catalytic activities in plasma, blood, and tumor were determined. The efficacy of repeat-dose compound with regard to tumor growth inhibition in vivo was also evaluated in the same MM xenograft models.

**Results:**

LC53-0110 is far more specific for the chymotrypsin-like proteolytic (β5) site of the 20S proteasome as compared to bortezomib, carfilzomib, or ixazomib. LC53-0110 treatment showed accumulation of ubiquitinated proteins, inhibited cell viability with a low nM range potency in various tumor cell lines, and showed potent activity on CD138^+^ cells isolated from MM patients who are resistant/refractory to current FDA-approved drug treatment. When a single dose was administered orally to tumor-bearing mice, LC53-0110 showed both greater maximum and sustained tumor proteasome inhibition as compared with ixazomib in MM xenograft models. The robust pharmacodynamic responses in tumor correlated with tumor growth regression. In addition, LC53-0151, an analog of LC53-0110, in combination with pomalidomide, a third-generation immunomodulatory drug, showed synergistic inhibition of tumor growth both in vitro and in the xenograft mouse model.

**Conclusions:**

In view of the in vitro, in vivo, and ex vivo profiles, further investigation of additional LC compounds in preclinical studies is warranted for the nomination of a clinical development candidate.

**Electronic supplementary material:**

The online version of this article (doi:10.1186/s12885-016-2285-2) contains supplementary material, which is available to authorized users.

## Background

The 26S proteasome is a multimeric complex consisting of a centrally-located 20S core catalytic complex flanked by 19S regulatory subunits [1–3]. The 19S caps contain multiple ATPase active sites and ubiquitin binding sites which recognize and unfold ubiquitinated protein substrates and transfer them to the 20S core. The 20S complex contains two outer α rings and two inner β rings. Each β ring contains three active enzymatic sites with distinct substrate specificities: chymotrypsin-like (CT-L) on the β5 subunit, trypsin-like (T-L) on the β2 subunit, and caspase-like (C-L) on the β1 subunit. The proteasome plays a critical role in cellular homeostasis by degrading a variety of proteins involved in cell cycle control, signal transduction, apoptosis, antigen processing, cell differentiation and proliferation through the ubiquitin-proteasome pathway. Therefore, the sites of the 26S proteasome, both 19S and 20S subunits, have been drug targets for various disease indications including cancer [4].

Bortezomib, a drug administered intravenously or subcutaneously, was the first proteasome inhibitor targeting enzymatic sites of the β chain and validated the proteasome as an anti-cancer target [5, 6]. It was approved for the treatment of mantle cell lymphoma and MM. Since FDA-approval of bortezomib, second-generation proteasome inhibitors with improved therapeutic indices have been pursued in various systems ranging from virtual modeling to tumor-bearing animals [7, 8]. Recently, carfilzomib also administered intravenously, was approved for the treatment of refractory MM [9–11]. There are currently other various small-molecule inhibitors in clinical development including ixazomib [12], oprozomib [13], and marizomib [14]. Whereas ixazomib and oprozomib are orally bioavailable proteasome inhibitors, marizomib is an intravenously administered inhibitor with less protease specificity.

Here, we describe the characterization of the preclinical pharmacology of LC53-0110 and its analog which are novel, reversible, selective, potent, and orally bioavailable proteasome inhibitors. We investigated the effects of LC-53-0110 on proteasome CT-L activities in vitro and in tumor cell lines, and addressed the anti-cancer mechanism by analyzing apoptosis pathways. Furthermore, we validated its effects on primary human myeloma cells of multiple myeloma patients. Finally, the compound’s in vivo effects were evaluated in multiple myeloma xenograft mouse models.

## Methods

### Human tumor cell lines and chemicals

Cell lines were obtained from the American Type Culture Collection (ATCC) and maintained in culture conditions at 37 °C under 5 % CO_2_. RPMI8226, MM.1S, U2932, Su-DHL-8, H1650, NCI-H1975, MDA-MB-231, and MCF-7 cells were maintained in RPMI-1640 (Gibco) containing 10 % fetal bovine serum (FBS, heat-inactivated; Gibco) and 1 % penicillin-streptomycin-amphotericin B (Gibco). HCT116 and HT-29 cells were maintained in McCoy’s 5A medium (Gibco) containing 10 % FBS and 1 % penicillin-streptomycin-amphotericin B.

Bortezomib, ixazomib, and carfilzomib were synthesized in our laboratory and the structures were confirmed by nuclear magnetic resonance. Pomalidomide was purchased from Tokyo Chemical Industry. Fluorogenic peptide substrates Suc-Leu-Leu-Val-Tyr-AMC (7-amido-4-methyl-coumarin) for the proteasomal CT-L activity, Z-Ala-Arg-Arg-AMC for the proteasomal T-L activity, and Z-Leu-Leu-Glu-AMC for the proteasomal C-L activity were purchased from Calbiochem, EMD Millipore.

### In vitro enzyme assays

RPMI8226 cell lysate was used for the in vitro proteasome inhibition assay. Cells were washed with PBS and resuspended in 20 mM Tris–HCl, pH 7.5. Then, cells were lysed on ice by dounce homogenization and the supernatants were collected by centrifugation. The assay was conducted in 96-well plates by incubating 2 μg of lysates with serial dilutions of a test compound and 20 μM of substrate at 37 °C for 1 h. After incubation, production of hydrolyzed AMC groups was measured using a FlexStation II Fluorometer (Molecular Devices) or using a Spectra MAX Gemini Fluorometer (Molecular Devices) with an excitation filter of 380 nm and an emission filter of 460 nm.

The non-proteasome protease panel assay was conducted at 10 μM of a test compound using standard substrate-based assays (GenScript, NJ). After 10 min incubation of proteases and inhibitor compounds at room temperature, substrates for respective proteases were added to initiate the reactions, and products from each reaction were analyzed with PHERAstar Plus (BMG LABTECH) or FlexStation 3 (Molecular Devices) using kinetics or endpoint model. Percent inhibition was calculated as [1- (sample activity-substrate control)/(enzyme activity-substrate control)]×100. The positive control of each non-proteasome protease showed 96–103 % inhibition, thereby validating the assay performance.

### Cell viability assays

Cell cytotoxicity was assessed using the ATP Bioluminescence Assay. RPMI8226, MM.1S, U2932, Su-DHL-8 cells were plated onto 96-well microtiter plates in culture medium and then treated with a serial dilution of compound in medium containing a final concentration of 0.5 % DMSO. Cultures were incubated for 48 h and cells were then assayed for viability using the CellTiter-Glo Luminescent Cell Viability Assay kit (Promega). For the adherent cell lines, cells were plated and incubated for 24 h. Cells were then treated with a serial dilution of compound for an additional 72 h and assayed for viability.

For the MTT [3-(4,5-dimethythiazol-2-yl)-2,5-diphenyltetrazolium bromide] assay, MM.1S cells were plated and incubated with DMSO or varying concentrations of LC53-0151 and pomalidomide. At the end of the incubation, the water soluble MTT was added and cell viability was determined by measuring optical density at 560 nm using SpectraMax M5e (Molecular Devices).

### Western immunoblot analysis

RPMI8226 cells were seeded and treated with either 500 nM of test compounds or 0.1 % DMSO. After incubation for 1 h, cells were washed with media three times, and incubated for an additional 4 or 24 h. Cells were washed with ice-cold PBS and lysed with Pro-Prep lysis buffer (iNtRON Biotechnology). Protein lysates were analyzed by Western immunoblotting using the following antibodies: anti-Ubiquitin (Enzo Life Sciences), anti-Hsp70 and anti-Hsp27 (Abcam), anti-Gadd34 (Proteintech), anti-caspase-8 (BD pharmingen), anti-β-Catenin, anti-p21, anti-phospho Hsp27 (S82), anti-caspase-9, anti-PARP, and anti-α-Tubulin (Cell Signaling). Images were obtained using ChemiDoc MP (Bio-Rad) and quantitation was done using Image Lab Software (Bio-Rad).

### Human primary cell samples

All human cell samples from MM patients and normal healthy donors were obtained after approval by the Institutional Review Board (IRB) for Human Research at the Catholic University of Korea and independent Ethics Review Board at the LG Life Sciences with written informed consent, respectively.

Primary MM cells were retrieved from bone marrow of patients and were enriched by Ficoll density centrifugation to a purity of >90 %, as necessary. Purity was assessed by cytomorphology on stained samples. CD138^+^ cells were collected using magnetic beads (Miltenyi Biotec) and plated at a density of 8 × 10^4^ cells per well onto 96-well plates in RPMI-1640 (WELGENE) containing 10 % FBS (WELGENE), 1 % penicillin-streptomycin-amphotericin B (Gibco), and 2 mM L-glutamine. Cells were then treated with a serial dilution of compound in medium containing a final concentration of 0.2 % DMSO. Cultures were incubated for 48 h and cells were then assayed for viability using the CellTiter-Glo Luminescent Cell Viability Assay kit (Promega).

Peripheral blood mononuclear cells (PBMCs) isolated from the heparinized blood samples of normal healthy donors were also plated and processed as described above.

### Tumor-bearing mouse studies

In vivo efficacy studies were performed in female BALB/c nude mice (8 weeks old) purchased from ORIENT Bio or female NOD.SCID mice (11–13 weeks old) purchased from HFK Bioscience. The animals were housed and maintained in a controlled environment including a 12 h light and 12 h dark cycle with automatic timers and received food and water *ad libitum*. All experiments were done under protocols approved by the Institutional Animal Care and Use Committee (IACUC). Moribund animals were sacrificed when necessary and animal carcasses were disposed of according to IACUC guidelines.

1 × 10^7^ MM.1S tumor cells in 0.1 ml of Dulbecco’s Phosphate-Buffered Saline (1:1 matrigel) were implanted subcutaneously into the right flank of female athymic nude mice. Tumor growth was monitored with calipers and mice were divided into homogeneous cohorts according to their initial tumor volume. Then within each cohort, mice were randomized to treatment groups just before dose initiation, thus ensuring that all the groups have comparable baselines. The compounds were formulated in 0.5 % methyl cellulose and administered at the indicated doses and regimens by oral gavage at a dose volume of 10 mL/kg. For the RPMI8226 model, a total of 1 × 10^7^ cells in 0.2 ml of PBS (1:1 matrigel) were implanted subcutaneously into the hind flank of female NOD.SCID mice. Whole blood was collected by cardiac puncture into tubes containing sodium heparin and cells were harvested by centrifugation. Blood cell pellets and tumor tissues were homogenized in lysis buffer (50 mM Tris–HCl, pH 8.0, 5 mM EDTA, 150 mM NaCl, 0.5 % Nonidet P-40, 0.5 mM phenylmethylsulfonyl fluoride, and 0.5 mM dithiothreitol) for 30 min at 4 °C. The lysates were centrifuged at 14,000 × g for 30 min, and the supernatants were collected as whole blood cell extracts and tumor cell extracts, respectively, for pharmacodynamic analysis. Blood, plasma, and tumor tissue samples were extracted with acetonitrile (0.1 % formic acid) containing internal standard, followed by centrifugation at 3600 rpm for 10 min. Supernatant was injected into the LC-MS/MS system for pharmacokinetic analysis. The mean, standard deviation (SD), and standard error of the mean (SEM) were calculated for each experimental group, and statistical analyses were performed using standard two tailed Student’s *t*-test (significance defined as *P* ≤ 0.05).

## Results

### LC53-0110 is a selective inhibitor of CT-L activity

**Fig. 1:**
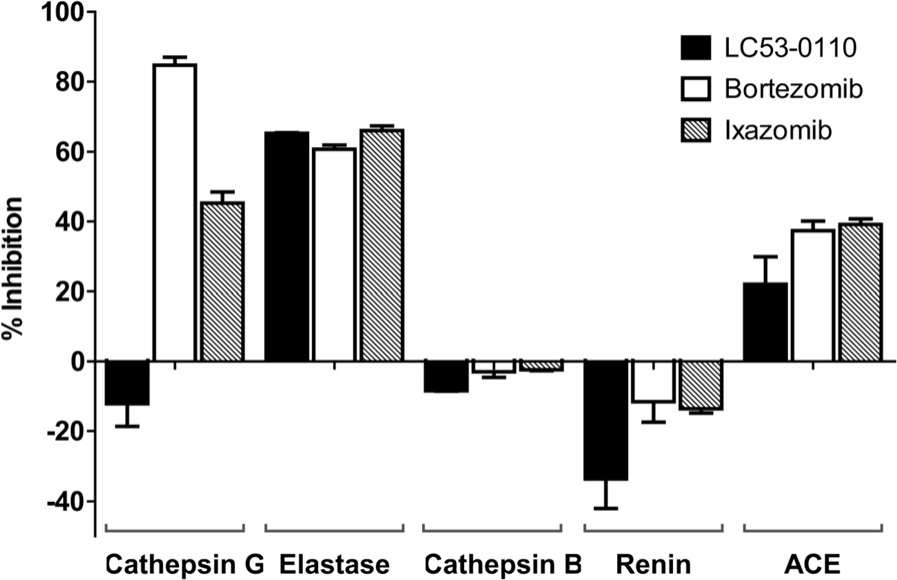
Inhibition of non-proteasomal proteases. 10 μM of proteasome inhibitor compounds were tested with a panel of proteases, Cathepsin G (serine protease with P1 specificity of phenylalanine/leucine/tyrosine), Elastase (neutrophil serine protease with P1 specificity of valine), Cathepsin B (cysteine protease), Renin (aspartate protease), and ACE (metalloprotease angiotensin converting enzyme). Percent inhibition was calculated based on compound’s effect on protease activity subtracted with a substrate control without an enzyme

LC53-0110 was identified following optimization of a new scaffold identified *in silico* for in vitro inhibition of proteasome CT-L activities using the fluorogenic substrate succinyl-Leu-Leu-Val-Tyr-AMC and human MM RPMI8226 cell lysates. LC53-0110 displayed potent inhibitory activity against CT-L, with IC_50_ values in the single nM range (Table 1). LC53-0110 was less potent against the other proteolytic sites of the 20S proteasome, i.e., T-L and C-L, with IC_50_ values of >20,000 nM and 600–1300 nM, respectively. When the relative potency of LC53-0110 was compared side by side with other proteasome inhibitors, LC53-0110 was found to be more specific toward CT-L inhibitory activity. LC53-0110 was determined to be a reversible inhibitor when CT-L activity was assessed in vitro at different time-points after RPMI8226 cells were pulse-treated for 1 h with the compound (data not shown). When assessed against representative non-proteasomal proteases (Fig. 1), LC53-0110 had relatively weak or no inhibitory activities except against neutrophil Elastase (serine protease with P1 specificity of valine), for which it showed 65 % inhibition at 10 μM, a potency similar to that of bortezomib and ixazomib. It is noteworthy that LC53-0110 differentiated itself from those proteasome inhibitors by showing no inhibitory activity against Cathepsin G (serine protease with P1 specificity of phenylalanine/leucine/tyrosine).

**Table 1:** Inhibition of the proteasome

Compound (route of administration)	Proteasome inhibition, IC_50_ (nM)			Binding kinetics
	CT-L, β5	T-L, β2	C-L, β1	
Bortezomib (IV/SC)	2 ~ 4	2400 ~ 3700	16 ~ 29	reversible
Carfilzomib (IV)	7 ~ 9	400 ~ 460	420 ~ 590	irreversible
Ixazomib (PO)	3 ~ 5	>10000	6 ~ 7	reversible
LC53-0110 (PO)	1 ~ 5	>20000	600 ~ 1300	reversible

The in vitro inhibition of the 20S proteasome catalytic activities was determined in RPMI8226 cellular lysates with fluorogenic peptide substrates specific for each catalytic subunit. Three reference compounds were tested in the assays along with LC53-0110. Binding kinetics was determined by assessing CT-L activity in RPMI8226 cells at different time points after 1-h pulse treatment of cells with a compound.

*CT-L* chymotrypsin-like, *T-L* trypsin-like, *C-L* caspase-like. *IV* intravenous, *SC* subcutaneous, *PO* per os, by mouth

### LC53-0110 inhibits viability in human tumor cell lines

When effects on cell viability were determined by bioluminescent measurement of cellular ATP, LC53-0110 inhibited viability in RPMI8226 cells with IC_50_ values of 6–12 nM at 48 h (Table 2). In additional tumor cell lines with diverse origins and genetic backgrounds, LC53-0110 was found to inhibit viability with IC_50_ values ranging from 3 nM to 25 nM, demonstrating potency in both hematologic and solid tumor cell lines tested.

**Table 2:** Inhibition of cellular viability

Cell line	Tumor type	IC_50_ (nM)
RPMI8226	Multiple myeloma	6 ~ 12
MM.1S	Multiple myeloma	3 ~ 6
U2932	Activated B-cell like diffuse large B-cell lymphoma	6 ~ 9
SU-DHL-8	Diffuse large B-cell lymphoma	11 ~ 18
HCT116	Colorectal carcinoma	13 ~ 14
HT-29	Colorectal adenocarcinoma	11 ~ 16
H1650	Lung carcinoma	7 ~ 22
NCI-H1975	Lung carcinoma	2 ~ 14
MDA-MB-231	Breast adenocarcinoma	8 ~ 14
MCF-7	Breast adenocarcinoma	14 ~ 25

The cytotoxic effects of LC53-0110 on a panel of tumor cell lines were determined by bioluminescent measurement of cellular ATP using CellTiter-Glo reagent. Hematologic and solid tumor cells were incubated with the compound for 48 h or 72 h, respectively prior to assay

**Fig. 2:**
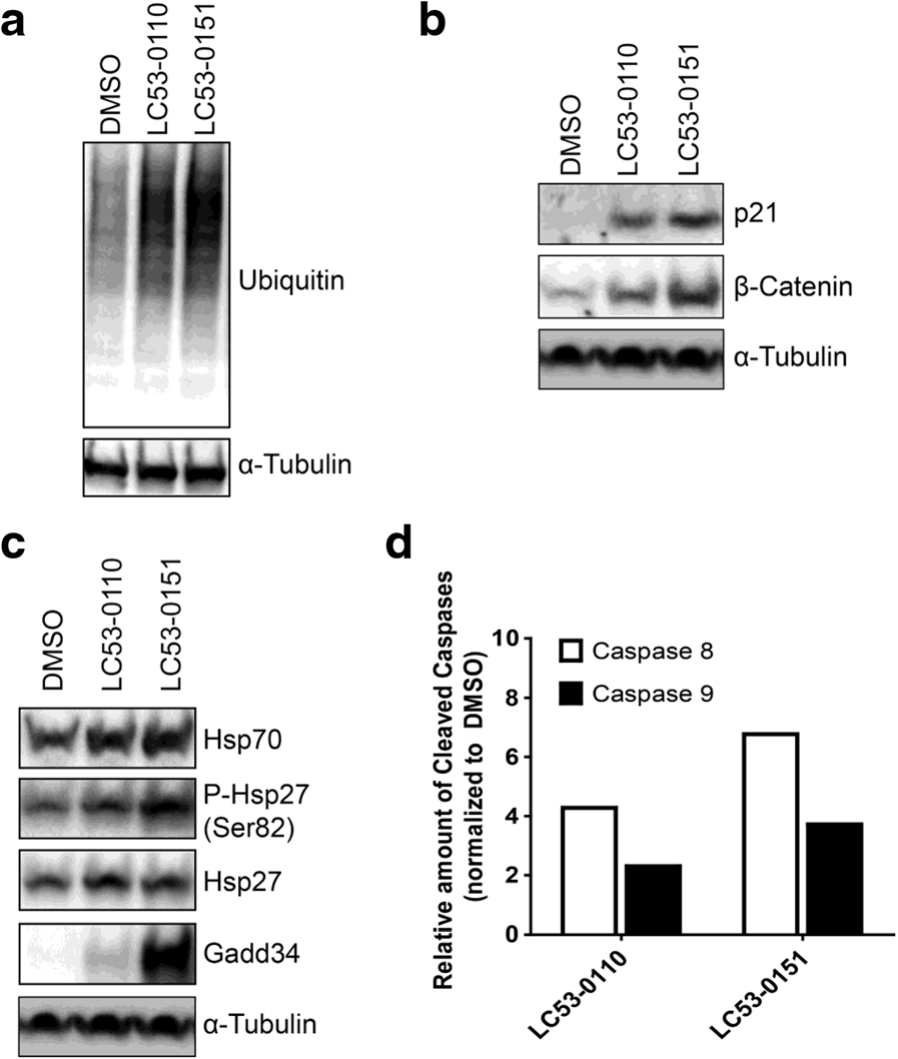
Accumulation of ubiquitinated proteins and activation of apoptosis in cells. Lysates were prepared from RPMI8226 cells 4 h (**a**) or 24 h (**b, c, d**) after a 1-h treatment with 0.1 % DMSO or 500 nM of proteasome inhibitors. Western blot analysis was done with anti-Ubiquitin, anti-p21, anti-β-Catenin, anti-Hsp70, anti-phospho Hsp27 (S82), anti-Hsp27, anti-Gadd34, anti-caspase-8, and anti-caspase-9 antibodies. α-Tubulin was used as a loading control. Cleaved caspase-8 and 9 bands on the western blot were quantitated and expressed as a fold-increase over DMSO control

For the assessment of the compound’s effect on the proteasome-dependent protein degradation pathway, cell lysates were prepared from RPMI8226 four hours after a one-hour pulse treatment with a compound and assessed by Western blot analysis with an ubiquitin-specific monoclonal antibody. The level of ubiquitinated proteins with a group of slower migrating bands in cells treated with LC53-0110 or its analog LC53-0151 were increased as compared to DMSO-treated cells (Fig. 2a), confirming inhibition of proteasome protease activities in cells, thereby blocking the proteasome-dependent polyubiquitinated protein degradation pathway. The inhibition of proteasome function was also confirmed by accumulation of the direct proteasome substrates p21 and β-Catenin (Fig. 2b).

Since accumulation of excessive or misfolded proteins triggers the cellular stress response including upregulation of heat shock protein (Hsp) expression and the unfolded protein response (UPR) [15–17], some of these components were examined to assess the mechanism of action of LC53-0110 and its analog LC53-0151. In RPMI8226 cells, significant basal levels of Hsp70, Hsp27, and phospho-Hsp27 were detected (Fig. 2c). Upon treatment with either compound, levels of Hsp70 and Hsp27 were increased, suggesting a collective cellular protection response by molecular chaperones. Phosphorylation of Ser82, one of the main phosphorylation sites of Hsp27 in vivo by MAP kinase-activated protein kinase 2 (MAPKAPK2) [18], was also increased, likely affecting its chaperone activity [19]. GADD34 was greatly induced upon compound treatment, suggesting UPR-dependent ER stress-induced cell death [15]. Therefore, to further address the mechanism of compound effects on cell viability, two types of caspases were assessed for activation of the apoptotic cell death pathway (Fig. 2d, Additional file 1: Figure S1A). Whereas caspase-8 triggers the apoptotic signal transduction cascade upon binding of death receptor ligands to their corresponding receptors, caspase-9 links induction of stress signaling pathways to the mitochondrial death pathway [20, 21]. Since activation of these caspases is associated with the cleavage of their inactive pro-enzyme forms, the level of cleaved caspases were determined in RPMI8226 cells. LC53-0110 and its analog LC53-0151 increased cleaved caspase-8 levels four to seven-fold and caspase-9 levels two to four-fold, respectively. Therefore, both compounds triggered mitochondria-dependent and -independent apoptotic cell death signaling pathways, thus leading to cleavage of the apoptotic marker poly(ADP-ribose) polymerase (PARP) (Additional file 1: Figure S1B). Treatment of human multiple myeloma MM.1S cells with either LC53-0110 or LC53-0151 also showed similar results (data not shown).

### LC53-0110 inhibits viability in primary human myeloma cells

**Fig. 3:**
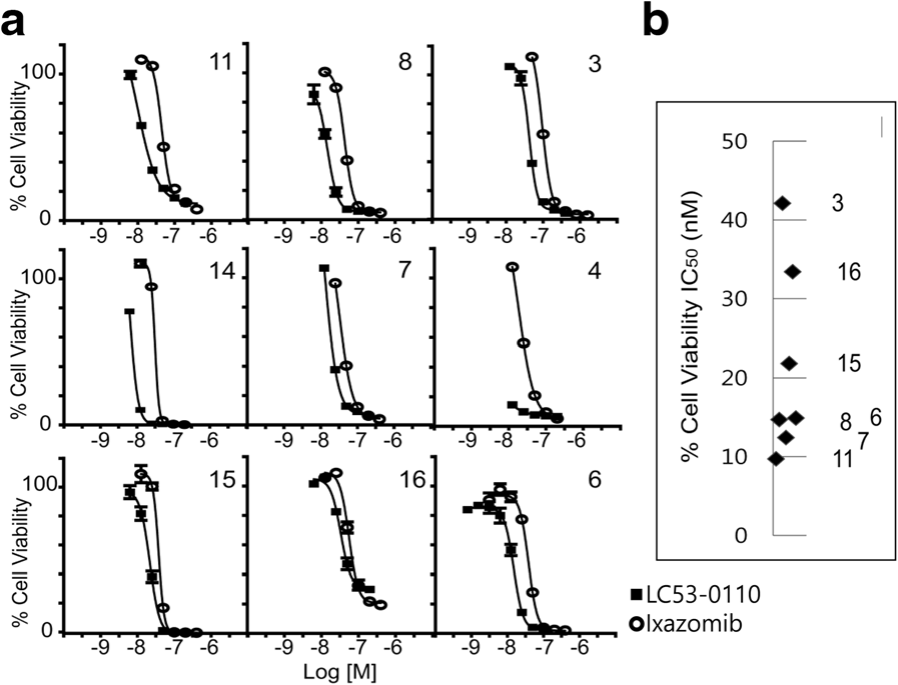
Inhibition of cellular viability in patient-derived samples. **a** CD138^+^ cells were purified from the bone marrow samples of MM patients (3, 4, 6–8, 11, 14–16). The cytotoxic effects of LC53-0110 (filled rectangle) and ixazomib (open circle) were determined by bioluminescent measurement of cellular ATP using CellTiter-Glo reagent after 48 h of treatment. Results are expressed as % cell viability over DMSO control. **b** The IC50 values for LC53-0110 treated MM patients

To assess effects of LC53-0110 on viability of primary human myeloma cells, CD138^+^ cells were purified from bone marrow samples of MM patients who had been either newly diagnosed or exposed to various standard therapies (Table 3). When effects on cell viability were determined by bioluminescent measurement of cellular ATP, the median IC_50_ for LC53-0110 and a range value were 15 nM and 32 nM, respectively (Fig. 3b, Additional file 2: Figure S2). In patient 4 and patient 14 samples where IC_50_s could not be determined due to ambiguous slopes, viability at 12.5 nM was 15 and 10 %, respectively (Fig. 3a). Therefore, regardless of prior therapy regimens applied to the patients and whether or not the patients became resistant or refractory, LC53-0110 showed a potent inhibition of viability in primary myeloma cells from the patients. The reference compound ixazomib was less potent than LC53-0110 in those patient samples (Fig. 3a). When normal PBMC samples from healthy donors were assessed for cytotoxicity, LC53-0110 and ixazomib showed more than 50 % viability at 10,000 nM, the highest concentration tested (data not shown). Therefore, normal PBMC cell viability was relatively less affected by proteasome inhibitors as compared with that of myeloma cells.

**Table 3:** Summary of multiple myeloma patients

Patient	Age	Type	D-S stage	V^R^	T^R^	L^R^	Number of treatment lines^a^
11	>65	IgG-λ	IIIa	N.A.	N.A.	N.A.	naive
4	>65	IgG-κ	IIIa	N.A.	N.A.	N.A.	naive
8	<65	λ	IIIa	Y	N.A.	Y	4
15	>65	IgA-λ	IIIa	Y	N.A.	Y	2
3	<65	IgG-λ	IIIa	Y	Y	Y	4
16	>65	IgG-κ	IIIa	Y	Y	Y	6
14	<65	λ	IIIb	Y	Y	Y	3
6	<65	IgG-κ	IIIa	Y	N	N.A.	2
7	<65	λ	IIIb	N	Y	Y	3

D-S Stage: The Durie-Salmon staging system. V: bortezomib. T: thalidomide. L: lenalidomide. R: resistance or refractory. N.A.: Not Applicable. Type: paraproteins IgG or IgA, κ-light chains κ or λ-light chains λ

^a^For patients who received autologous stem cell transplantation (ASCT), a regimen of induction + ASCT+/−maintanence was counted as one treatment line. Naive: no past treatment

### LC53-0110 strongly inhibits proteasome catalytic activity in tumor xenograft models

**Fig. 4:**
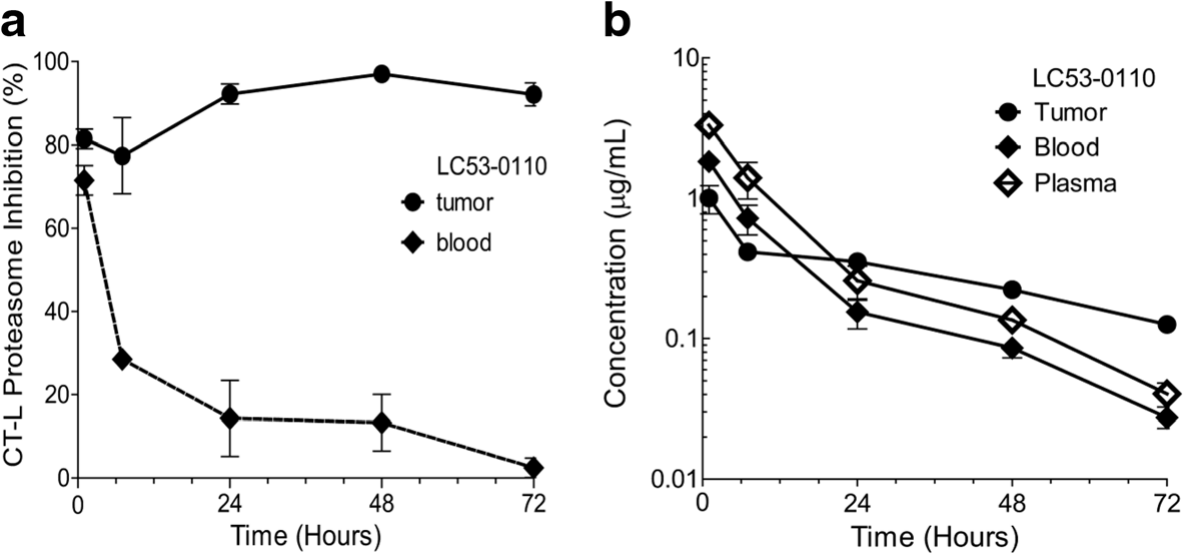
PD and PK profiles of LC53-0110 in mice bearing human multiple myeloma xenografts. **a** MM.1S tumor bearing mice were administered orally with a single dose of LC53-0110 at 50 mg/kg, and the 20S proteasome catalytic activities in tumor and blood were evaluated with CT-L subunit-specific fluorogenic peptide substrates. Data points, mean +/−SE (*n* = 3). **b** In the same study, concentration-time profiles of LC53-0110 in plasma, blood, and tumor were determined. Data points, mean +/−SD (*n* = 3)

Having established anti-viability in tumor cell lines, systemic effects of LC53-0110 on tumor growth in an organism were assessed. MM.1S xenograft tumors were subcutaneously implanted in the hind flank of athymic nude mice and the ability of LC53-0110 to inhibit endogenous proteasomal CT-L activity was examined following a single oral dose of 50 mg/kg. The tumors were harvested 1, 7, 24, 48 or 72 h after treatment and homogenized in lysis buffer. Blood was collected at the same time as tumor tissues were harvested, and blood lysates were prepared as well. The inhibition of proteasomal CT-L catalytic activity in the tumors was 82 % at 1 h, reached a maximum of 97 % at 48 h, and persisted through 72 h (Fig. 4a). In contrast, the inhibition of CT-L catalytic activity in the blood decreased from 72 % at 1 h to 29 % at 7 h, and further decreased with only minimal or no inhibition evident by 72 h.

The concentrations of LC53-0110 in plasma, blood, and tumors were also determined in this study (Fig. 4b). Tumor concentrations were 1 μM at 1 h and decreased to 0.4 μM at 7 h and 24 h. Then, the compound levels gradually decreased to 0.1 μM by 72 h. In contrast, blood concentrations were 1.8 μM at 1 h and continuously decreased to 0.03 μM by 72 h. The slope of concentration change in plasma over time was similar to that in blood with 3.3 μM at 1 h and 0.04 μM by 72 h. Therefore, whereas 97 % inhibition of tumor CT-L activity occurred at the tumor concentration of 0.2 μM, 14 % inhibition of blood CT-L activity occurred at the blood concentration of 0.16 μM. The data presented here show the pharmacodynamic effect of LC53-0110 to be greater in tumors over blood as well to outlast a detectable plasma or blood exposure.

### LC53-0110 displays robust anti-tumor activity in tumor xenograft models

**Fig. 5:**
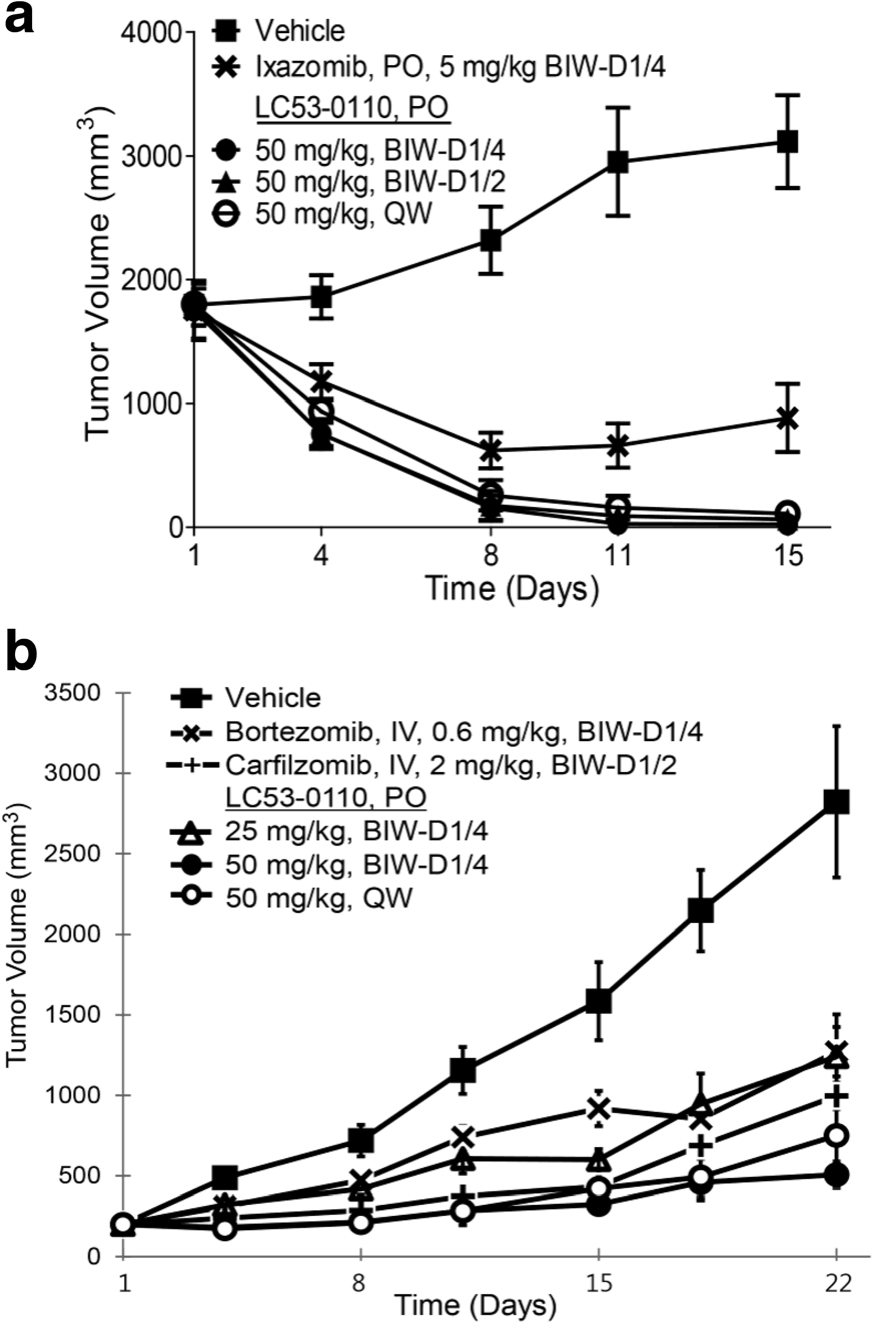
Anti-tumor activity of LC53-0110 in mice bearing human multiple myeloma xenografts. **a** The LC and reference compounds were orally administered twice a week (BIW) or once a week (QW) in mice bearing MM.1S xenografts as indicated. The mean tumor volume for each animal in the vehicle and treatment groups was determined during the study. Data points, mean+/−SE (*n* = 6). **b** LC53-0110 was orally administered twice a week or once a week in mice bearing RPMI8226 xenografts as indicated, and reference compounds were intravenously administered twice a week. The mean tumor volume for each animal in the vehicle and treatment groups was determined during the study. Data points, mean+/−SE (*n* = 8)

The efficacy of repeat-dose LC53-0110 with regard to tumor growth inhibition in vivo was evaluated in the previously described MM.1S as well as RPMI8226 xenograft models. Due to its durable target modulation activity, i.e., a sustained pattern of proteasomal CT-L inhibition in tumors, it could be predicted that dosing every several days or even once a week with LC53-0110 might result in significant anti-tumor efficacy in vivo. Indeed, when administered orally at 50 mg/kg of LC53-0110 (Fig. 5a), MM.1S tumor regression was observed on day 4 post-dosing (42–52 % of start tumor volume) and almost complete tumor regression was achieved by day 15 with all dosing regimens tested (twice or once a week schedules). In contrast, when orally administered twice a week at 5 mg/kg, ixazomib showed a maximum tumor regression on day 8 (36 % of start tumor volume), after which the tumors appeared to regrow, reaching 58 % of start tumor volume and 72 % tumor growth inhibition on day 15 as compared to the vehicle control group.

The strong efficacy of LC53-0110 was also evident in the RPMI8226 xenograft model. Dose-dependent tumor growth inhibition was achieved by LC53-0110 at 25 mg/kg (56 %) and 50 mg/kg (82 %) on day 22 when orally administered twice a week (Fig. 5b). Tumor growth inhibition by LC53-0110 dosed 50 mg/kg once a week was similar to that seen with the twice a week dosing regimen throughout the treatment period. Moreover, tumor growth inhibition by 50 mg/kg LC53-0110 administered orally was greater than 0.6 mg/kg bortezomib or 2.0 mg/kg carfilzomib, both of which were administered intravenously. The anti-tumor effect of LC53-0110 as assayed by tumor volume change was also confirmed by weight measurement of tumors extracted after final tumor volume measurement in tumor-bearing mice (data not shown).

### An analog of LC53-0110 synergizes with an immunomodulatory drug for anti-tumor activity

**Fig. 6:**
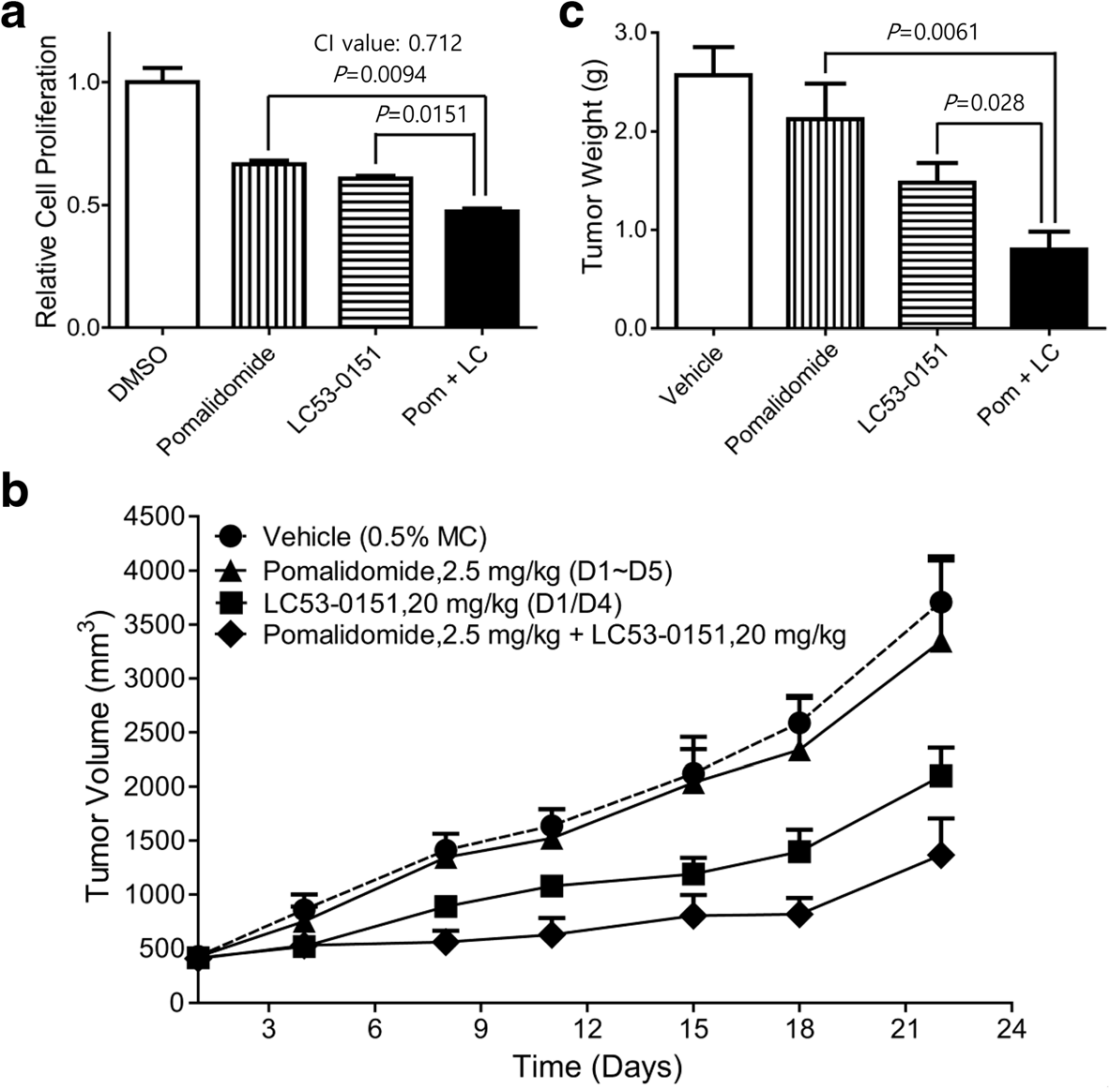
Anti-tumor activity of an LC53-0110 analog combined with an immunomodulatory drug. **a** The cytotoxic effects of 5 nM of LC53-0151 or 2 μM of pomalidomide alone or together on MM.1S cells were determined by MTT assay. **b** LC53-0151 or pomalidomide alone or together in mice bearing MM.1S xenografts as indicated. The mean tumor volume for each animal in the vehicle and treatment groups was determined during the study. **c** The mean tumor weight for each animal in the vehicle and treatment groups was determined at the end of the study. Data points, mean+/−SE (*n* = 8)

To address if an LC compound enhances anti-tumor activity when combined with immunomodulatory drugs, MM.1S cells were treated with pomalidomide, a third-generation immunomodulatory drug, for 24 h and then with the LC53-0110 analog LC53-0151 for an additional 24 h across a range of concentrations. When cell proliferation was measured by MTT analysis, cells treated with LC53-0151 or pomalidomide as a single agent showed lower viability as compared to DMSO-treated control cells, and the combination of both agents showed a greater inhibition of viability than either agent alone (Fig. 6a). Moreover, isobologram analysis confirmed that LC53-0151 and pomalidomide induced synergistic cytotoxicity in MM.1S cells (combination index analyzed by CalcuSyn S/W < 1.0).

In order to address if the observed synergism is also displayed in vivo, the efficacy of oral repeat-dose LC53-0151 was evaluated with regard to tumor growth inhibition in MM.1S xenografted nude mice with or without pomalidomide treatment. LC53-0151 and pomalidomide were orally administered for 3 weeks at 20 mg/kg twice a week and at 2.5 mg/kg for 5 days per week, respectively. Whereas pomalidomide treatment displayed 10 % reduction of tumor volume with no statistically significance as compared to vehicle-treated mice, LC53-0151 treatment exhibited 43 % reduction of tumor volume (Fig. 6b). In addition to its anti-tumor efficacy as a monotherapy, LC53-0151 potentiated the anti-tumor efficacy of pomalidomide, resulting in a 63 % reduction of tumor volume on day 22 as compared to vehicle-treated mice. The improved efficacy of LC53-0151 when combined with pomalidomide was also confirmed by weight measurement of tumors extracted after final tumor volume measurement in tumor-bearing mice (Fig. 6c).

## Discussion

Since bortezomib came on the market, there has been significant improvement in survival of MM patients [6]. However, the drug has some drawbacks such as toxicities including gastrointestinal symptoms (e.g., diarrhea), fatigue, thrombocytopenia, anemia, and peripheral neuropathy (PN), which is a common complication [22, 23]. In addition, it must be administered by intravenous or subcutaneous injection. Carfilzomib, another proteasome inhibitor on the market, has a low incidence of PN, but it has common serious adverse events (AEs) including fever, pneumonia, acute renal failure, and congestive heart failure, and it also must be administered by intravenous injection [24–27]. Since proteasome inhibitors emerged as an important component of MM treatment, logistical challenges for those injection approaches have become clear, especially for patients who need long-term therapy. Recent studies of the investigational oral proteasome inhibitor ixazomib, an improved version of bortezomib, showed a lower incidence of grade 3 PN as compared to bortezomib, and drug-related other grade 3 AEs including diarrhea, fatigue, thrombocytopenia, neutropenia, and skin rash [28–33]. With improving survival in MM, the oral route of administration of ixazomib is considered to be high value, especially with the all-oral triple combination of ixazomib, an immunomodulatory agent, and a steroid being the most convenient and effective [34]. This is a particularly important factor for post-transplant maintenance, which can last several years. Therefore, we focused on developing an oral proteasome inhibitor that can be differentiated from ixazomib on the efficacy and safety profiles.

LC53-0110 is a more specific inhibitor of CT-L as compared to ixazomib, which has a similar potency toward C-L as CT-L (Table 1). The biological role of the individual proteasomal active sites is not fully understood. No phenotypic or proteolytic defects were observed upon inactivation of C-L sites by site-directed mutagenesis of catalytic threonines in the yeast *S. cerevisiae* [35, 36]. Using chemical inhibitors or activity-based probes of C-L site, there have been some efforts to address the question of whether inhibition of this site is important for anti-tumor activity, especially in conjunction with inhibition of CT-L site [37–40]. However, due to the properties of the selective compounds including potency, cell permeability, stability, ADME/PK, and toxicity, the physiological consequences of C-L inhibition still need to be established. The study with carfilzomib, a relatively specific CT-L inhibitor like LC53-0110, supported the notion that inhibition of cellular CT-L activity is sufficient to kill hematological tumor cells, in particular MM cells, with minimal effects on normal leukocytes [41]. This is consistent with the findings in our study with LC53-0110, i.e., accumulation of ubiquitinated proteins, decrease of cell viability, and increase of apoptosis in MM cells (Table 2, Fig. 2).

LC53-0110 with CT-L specificity also showed potent inhibition of viability of MM cells obtained from patients in a dose-dependent manner (Fig. 3). Although it is risky to make a conclusion with a limited number of patients, patients who were newly diagnosed (patients 11 and 4) showed the lower end of cellular potency (i.e., most potent) as compared to those patients with multiple prior therapies (2–6 regimens) including proteasome inhibitors, thalidomide, lenalidomide, dexamethasone, histone deacetylase inhibitor, and chemotherapeutic drugs. Interestingly, LC53-0110 showed increased potency in MM cells obtained from bortezomib- and ixazomib-refractory patient with 10 % viability at 12.5 nM, at which ixazomib did not show any effect on viability (patient 14, Fig. 3a). LC53-0110 clearly showed the ability to inhibit viability of MM cells of patients who were progressed after various treatments, with potency similar to those of MM cell lines. Therefore, it is likely that the proteasome CT-L active sites inhibited by LC53-0110 were not affected in these patients.

In human MM xenograft models in nude mice, oral administration of LC53-0110 showed much greater proteasome inhibition in tumor compared with blood, which was also the case for ixazomib [42] (Fig. 4a; and data not shown). However, LC53-0110 showed greater maximum and sustained tumor proteasome inhibition as compared with ixazomib (>90 % with a duration of action of at least 72 h). On repeat dosing, LC53-0110 showed complete tumor growth regression in MM.1S xenografts, a greater effect as compared with ixazomib (Fig. 5a). The efficacious activity of LC53-0110 was also demonstrated by the lack of measurable tumor mass even six weeks after the final dose in the surviving mice (data not shown). LC53-0110 also showed greater tumor growth inhibition than bortezomib and carfilzomib in RPMI8226 xenografts at well-tolerated doses (Fig. 5b). However, in this efficacy model, all 8 mice died or were euthanized due to moribund condition the day after the first oral treatment with ixazomib at 5 mg/kg, which most likely is related to the possible toxicity of ixazomib.

Various agents have been proven to enhance anti-tumor activity in combination with other agents, and the addition of immunomodulatory drugs such as thalidomide or lenalidomide is considered one of the standard MM therapies [43–48]. Recently, treatment of relapsed MM with the proteasome inhibitor carfilzomib in combination with lenalidomide and dexamethasone showed better progression-free survival and overall response than lenalidomide and dexamethasone alone [49]. Pomalidomide is a new generation of immunomodulatory drug which was shown to confer a survival advantage even in patients who are refractory to both bortezomib and lenalidomide [50]. Pomalidomide is currently being investigated in combination with dexamethasone [51, 52], with bortezomib and dexamethasone [53], or with carfilzomib and dexamethasone [54] in patients with relapsed/refractory MM. LC53-0151, an analog of LC53-0110, in combination with pomalidomide also showed further synergistic inhibition of tumor growth both in vitro and in the xenograft mouse model (Fig. 6).

## Conclusions

The orally available LC53-0110 and its analog LC53-0151 having strong efficacy and toxicity profiles may further improve the therapeutic efficacy, safety, and convenience for patients when used in combination with orally available immunomodulatory drugs. Therefore, in view of the in vitro, in vivo, and ex vivo profiles, further investigation of additional LC compounds in preclinical studies is warranted for the nomination of a clinical development candidate.

## Additional files

Additional file 1: Figure S1. Activation of apoptosis in cells. Lysates were prepared from RPMI8226 cells 24 h after a 1-h treatment with 0.1 % DMSO or 500 nM of proteasome inhibitors. Western blot analysis was performed with anti-caspase-8 and anti-caspase-9 antibodies (A), or anti-PARP antibody (B). α-Tubulin was used as a loading control. Fl: full-length, Cl: cleaved. (PDF 31 kb) Additional file 2: Figure S2. Inhibition of cellular viability in patient-derived samples. CD138^+^ cells were purified from the bone marrow samples of MM patients (3, 4, 6–8, 11, 14–16). The cytotoxic effects of LC53-0110 were determined by bioluminescent measurement of cellular ATP using CellTiter-Glo reagent after 48 h of treatment. Results are expressed as % cell viability over DMSO control. Cell viability was plotted for all LC53-0110 treated multiple myeloma patients. Data points, mean+/−SE. (PDF 41 kb)

## Abbreviations

C-L: caspase-like
CT-L: chymotrypsin-like
MM: multiple myeloma
T-L: trypsin-like
