# Supplementary figures and images for: Oral proteasome inhibitor with strong preclinical efficacy in myeloma models
Source: BMC Cancer. 2016 Mar 24;16:247. doi: 10.1186/s12885-016-2285-2 (PMC4806471; doi:10.1186/s12885-016-2285-2)

Figure S1

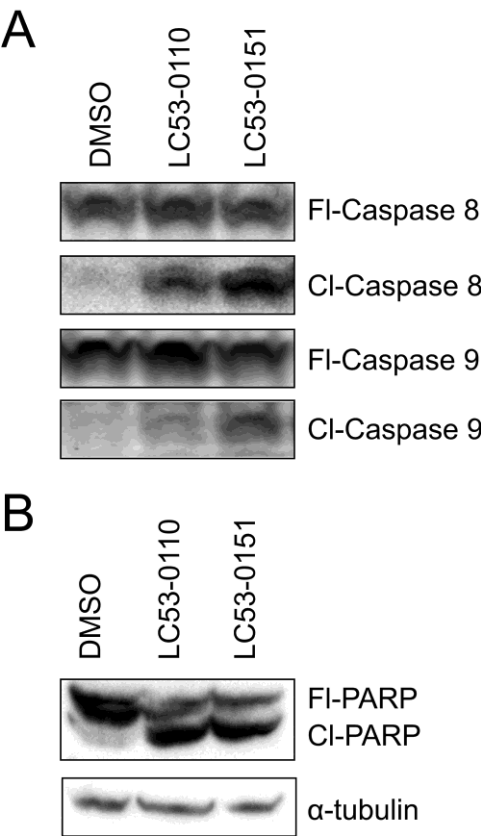

Supplement: Additional file 1: Figure S1. — Activation of apoptosis in cells. Lysates were prepared from RPMI8226 cells 24 h after a 1-h treatment with 0.1 % DMSO or 500 nM of proteasome inhibitors. Western blot analysis was performed with anti-caspase-8 and anti-caspase-9 antibodies (A), or anti-PARP antibody (B). α-Tubulin was used as a loading control. Fl: full-length, Cl: cleaved. (PDF 31 kb) [file 12885_2016_2285_MOESM1_ESM.pdf]

Figure S2

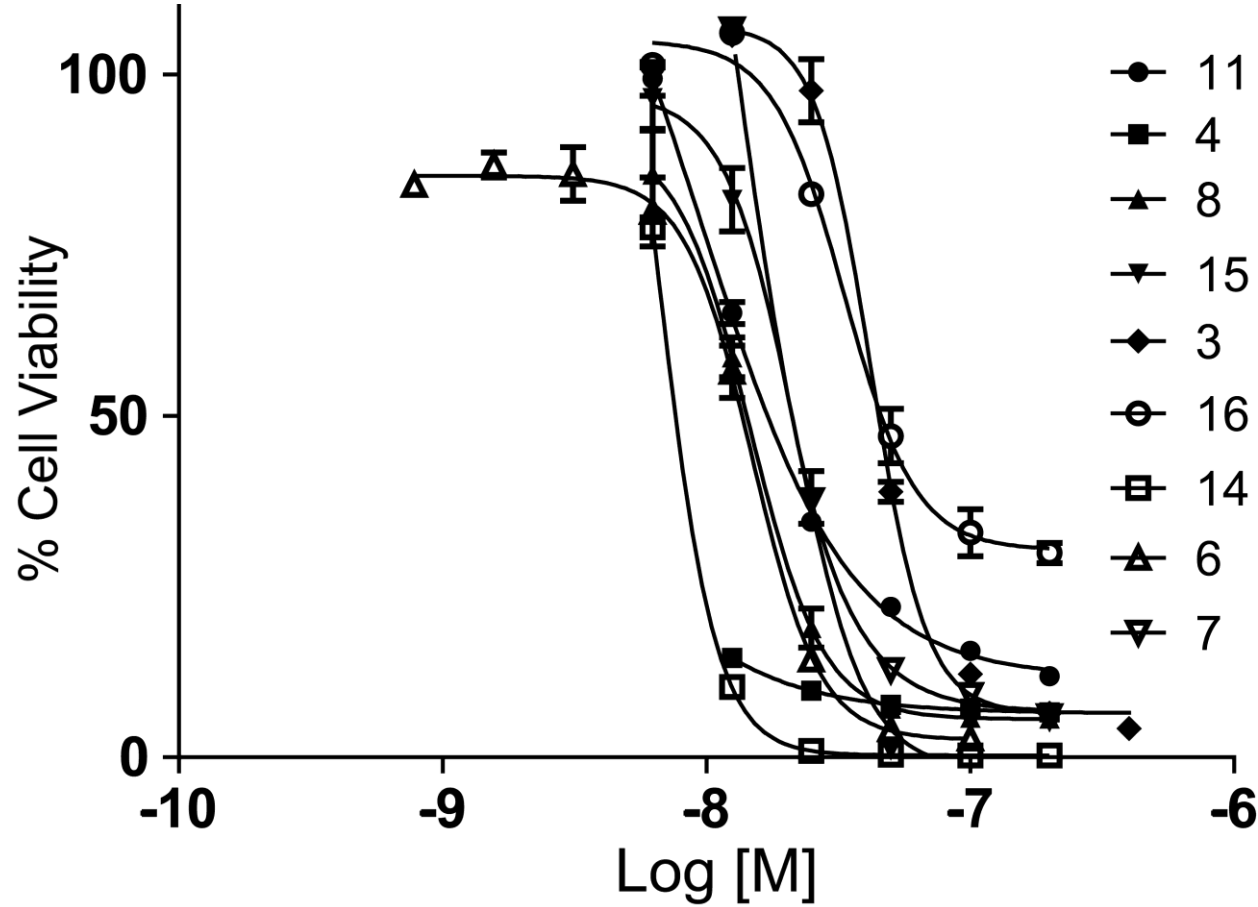

Supplement: Additional file 2: Figure S2. — Inhibition of cellular viability in patient-derived samples. CD138+ cells were purified from the bone marrow samples of MM patients (3, 4, 6–8, 11, 14–16). The cytotoxic effects of LC53-0110 were determined by bioluminescent measurement of cellular ATP using CellTiter-Glo reagent after 48 h of treatment. Results are expressed as % cell viability over DMSO control. Cell viability was plotted for all LC53-0110 treated multiple myeloma patients. Data points, mean+/−SE. (PDF 41 kb) [file 12885_2016_2285_MOESM2_ESM.pdf]
